# Erythroplasia of Queyrat treated with topical 5-fluorouracil*

**DOI:** 10.1590/abd1806-4841.20164595

**Published:** 2016

**Authors:** João Roberto Antônio, Carlos Roberto Antônio, Lívia Arroyo Trídico, Fernanda Tomé Alves, Ivan Rollemberg

**Affiliations:** 1 Faculdade de Medicina de São José do Rio Preto (FAMERP) - São José do Rio Preto (SP), Brazil

**Keywords:** Carcinoma in Situ, Penis, Therapeutics

## Abstract

We report a 33-year-old male patient diagnosed with erythroplasia of Queyrat. The patient had an erythematous and eroded lesion affecting more than 50% of the glans associated with bleeding and local pain. Despite previous indication of penectomy, he was successfully treated with topical 5-fluorouracil.

## INTRODUCTION

Erythroplasia of Queyrat (EQ) is a rare in situ squamous cell carcinoma (SCC) of the glans penis, which typically appears as one or more well-marginated erythematous velvety plaques.^1^ Its cause is still unknown.^2^ It affects almost exclusively uncircumcised young men. Predisposing factors include lack of hygiene, smegma, humidity, and heat.^3^ For this reason, early circumcision is considered an appropriate preventive measure for male patients.^3^ The role of human papillomavirus (HPV) - oncogenic types 8, 39, 51, and mostly 16 - was recently proposed in its pathogenesis.^4^ Studies estimate progression to invasive SCC in a third of patients.

Histopathological examination (hematoxylin-eosin stain) shows identical results to Bowen's disease. However, the epidemiology and anatomical location of these two conditions are mutually exclusive.^5^ Similar to Bowen's disease (BD), the presence of infiltration, nodularity, and/or ulceration often suggests a possible conversion to an invasive squamous cell carcinoma.^6^

As EQ has a strong tendency to develop into invasive penile carcinoma and treatment options include tissue removal - either by invasive techniques, such as partial or total penectomy, or through noninvasive techniques, such as laser ablation, cryosurgery, photodynamic therapy, topical 5-fluorouracil, and imiquimod 5%.^1,2^ When selecting the most appropriate therapy, it is important to accurately determine, by means of biopsy, whether the patient has EQ or invasive SCC.^7^

The removal of cancer by partial or total penectomy is a standard therapy, but these radical procedures can cause considerable mental distress, including suicide. Therefore, the development of a non-invasive alternative treatment for EQ is essential. We report the case of an EQ patient with penectomy indicated by an urologist, but who responded well to topical treatment with topical 5-fluorouracil.

## CASE REPORT

We report a 33-year-old male patient present to our institution with a history of having performed circumcision for about eight years due to an eroded lesion on the distal glans treated with antibiotics and topical corticosteroids without improvement. The patient reported that the lesion had been persistent for eight years and had recently increased in size. Local pain and possible bleeding were also reported. The patient had gone through prior consultation with a urologist who suggested radical excision of the tip of the glans.

**Figure 1:**
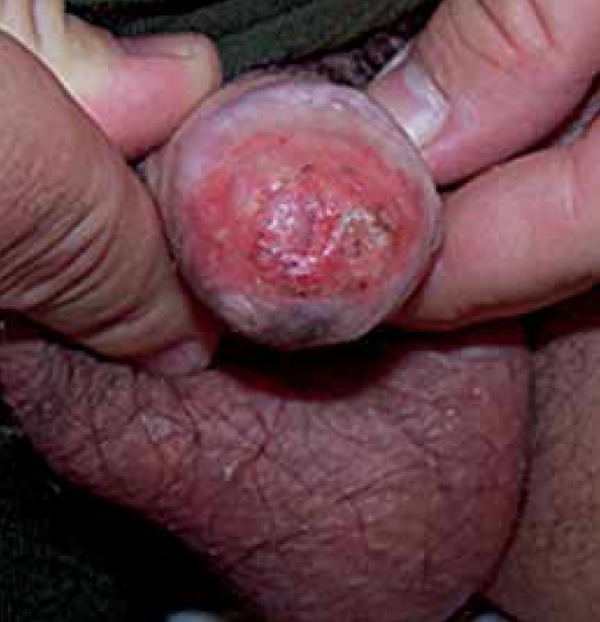
Initial lesion affecting more than 50% of the glans

Dermatological physical examination revealed eroded erythematous flat lesions, with sharp and regular edges, affecting more than 50% of the glans (Figure 1). We observed no inguinal lymphadenopathy. General physical examination was normal.

**Figure 2:**
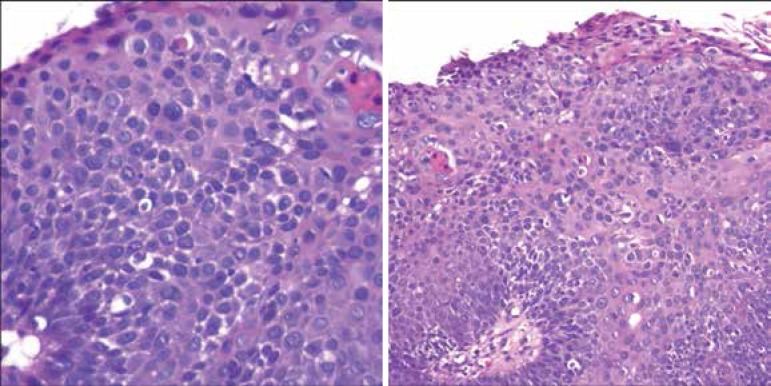
Initial lesion affecting more than 50% of the glans

An incisional biopsy showed proliferation of atypical keratinocytes in the epidermis, with perivascular lymphocytic infiltrate in the adjacent dermis compatible with EQ (Figure 2).

**Figure 3:**
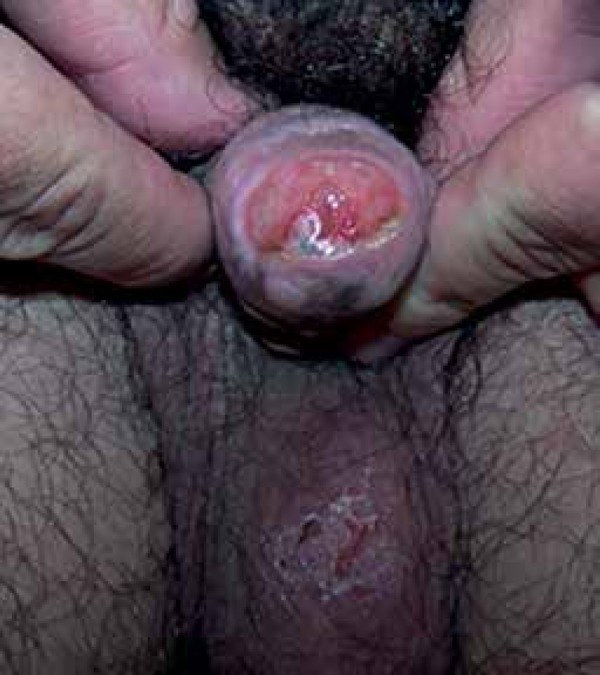
Fifteen days after the first application of 5-fluorouracil 5% twice daily for two weeks

**Figure 4:**
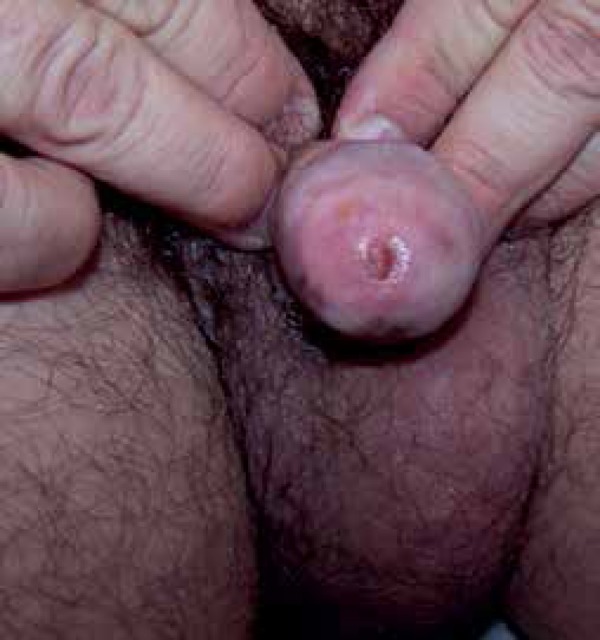
Fifteen days after the third application of 5-fluorouracil 5%

**Figure 5:**
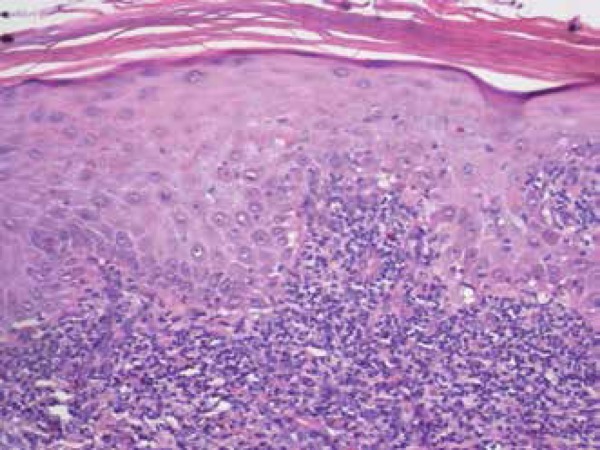
Biopsy performed two months after the end of treatment: standard lichenoid interface dermatitis with no atypical cells in the epidermis

Given this diagnosis and the patient's age, we chose to perform a nonsurgical treatment with topical 5-fluorouracil 5%. Initially, 5-fluorouracil 5% was applied to the entire lesion twice daily for two weeks. We obtained a favorable result fifteen days after the application, considering the initial irritation predicted with the use of this medication (Figure 3). We repeated two more cycles of treatment for two weeks, with an interval of fifteen days between the cycles (Figure 4). Thirty days after the third cycle, the lesion showed complete clinical resolution, displaying only discreet erythema at the distal glans. Two months after the third cycle, we performed a biopsy that showed standard lichenoid interface dermatitis with no atypical cells in the epidermis (Figure 5).

**Figure 6:**
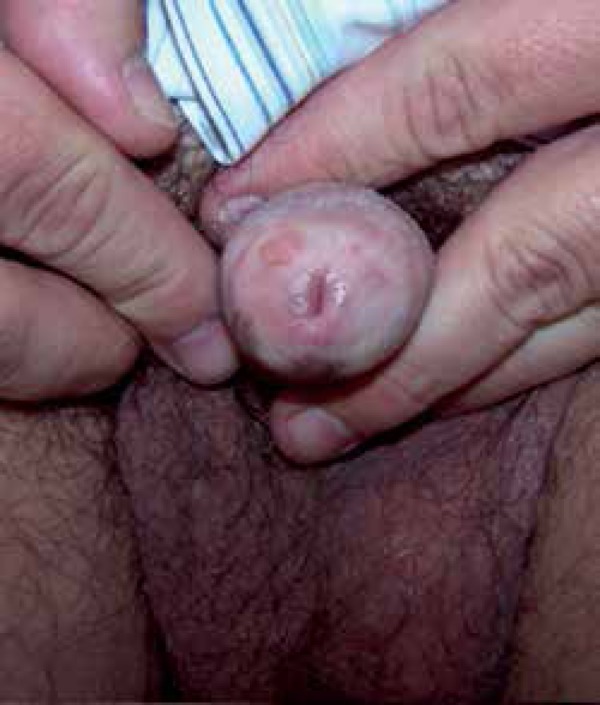
Scar, six months after the end of treatment

Thus, we decided to monitor the patient monthly. Six months after the treatment, physical examination revealed only a scar in the lesion site (Figure 6). One year after the treatment, the patient showed no recurrence, progressing to complete healing and maintaining continuous follow up.

## DISCUSSION

Since about 33% of EQ cases become invasive - and the risk of metastases to regional lymph nodes becomes relevant when the tumor invades the submucosa - standard treatment options are usually aggressive.^8,9^

Surgical treatment involving partial or total penectomy should be performed with caution, considering that it is often accompanied by psychological distress, intense emotional distress, and depression.^6^ Thus, the biopsy of the lesion is essential in the therapeutic decision. Physicians must assess the degree of cellular atypia to check if it is restricted to the epidermis, differing EQ from invasive SCC.^1,7^

In a review paper on the treatment of EQ, Goette concluded that a therapeutic regimen with 5-fluorouracil is associated with high cure rates without evidence of recurrence.^10^ 5-fluorouracil acts as an antimetabolite by inhibiting the synthesis of DNA and RNA. The drug can be used continuously for 4-5 weeks and its side effects include erythema, edema, and local irritation.^10^ In the present case, we used a two-week treatment cycle because the patient presented significant local irritation at the end of the second week, which resolved in the interval between the cycles.

By opting for conservative treatment with 5-fluorouracil, continued and prolonged clinical follow-up is fundamental. The results of every therapeutic step should be monitored and attention should be given to the possible evolution of the lesion to invasive SCC.
